# Author Correction: Induced unconventional superconductivity on the surface states of Bi_2_Te_3_ topological insulator

**DOI:** 10.1038/s41467-017-02594-x

**Published:** 2018-01-05

**Authors:** Sophie Charpentier, Luca Galletti, Gunta Kunakova, Riccardo Arpaia, Yuxin Song, Reza Baghdadi, Shu Min Wang, Alexei Kalaboukhov, Eva Olsson, Francesco Tafuri, Dmitry Golubev, Jacob Linder, Thilo Bauch, Floriana Lombardi

**Affiliations:** 1 Department of Microtechnology and Nanoscience, Chalmers University of Technology, SE-41296 Göteborg, Sweden; 2 Institute of Chemical Physics, University of Latvia, 19 Raina Boulevard, LV-1586 Riga, Latvia; 3 Shanghai Institute of Microsystem and Information Technology, Chinese Academy of Sciences, 865 Changning Road, Shanghai, CN-200050 China; 4 Department of Applied Physics, Chalmers University of Technology, SE-41296 Göteborg, Sweden; 5 Dipartimento di Fisica E. Pancini, Università di Napoli Federico II, IT-80126 Napoli, Italy; 6 CNR-SPIN Institute of Superconductors, Innovative Materials and Devices, IT-80125 Napoli, Italy; 7 Department of Applied Physics, Aalto University School of Science, P.O. Box 13500, Aalto, FI-00076 Finland; 8 Department of Physics, QuSpin Center of Excellence, Norwegian University of Science and Technology, N-7491 Trondheim, Norway

**Keywords:** Topological matter, Superconducting devices, Superconducting properties and materials

Correction to: *Nature Communications* 10.1038/s41467-017-02069-z, published online 08 December 2017

The original version of this Article omitted the following from the Acknowledgements:

“This work was partly supported by the Research Council of Norway through its Centres of Excellence funding scheme, project number 262633, QuSpin.”

This has now been corrected in both the PDF and HTML versions of the article.

