# Author Correction: Induced unconventional superconductivity on the surface states of Bi_2_Te_3_ topological insulator

**DOI:** 10.1038/s41467-018-02945-2

**Published:** 2018-01-30

**Authors:** Sophie Charpentier, Luca Galletti, Gunta Kunakova, Riccardo Arpaia, Yuxin Song, Reza Baghdadi, Shu Min Wang, Alexei Kalaboukhov, Eva Olsson, Francesco Tafuri, Dmitry Golubev, Jacob Linder, Thilo Bauch, Floriana Lombardi

**Affiliations:** 1 Department of Microtechnology and Nanoscience, Chalmers University of Technology, SE-41296 Göteborg, Sweden; 2 Institute of Chemical Physics, University of Latvia, 19 Raina Boulevard, Riga, LV-1586, Latvia; 3 Shanghai Institute of Microsystem and Information Technology, Chinese Academy of Sciences, 865 Changning Road, CN-200050 Shanghai, China; 4 Department of Applied Physics, Chalmers University of Technology, SE-41296 Göteborg, Sweden; 5 Dipartimento di Fisica E. Pancini, Università di Napoli Federico II, IT-80126 Napoli, NA Italy; 6 CNR-SPIN Institute of Superconductors, Innovative Materials and Devices, IT-80125 Napoli, NA Italy; 7 Department of Applied Physics, Aalto University School of Science, P.O. Box 13500, FI-00076 Aalto, Finland; 8 Department of Physics, QuSpin Center of Excellence, Norwegian University of Science and Technology, N-7491 Trondheim, Norway

**Keywords:** Topological matter, Superconducting devices, Superconducting properties and materials

Correction to: *Nature Communications* 10.1038/s41467-017-02069-z, published online 08 Dec 2017

The original version of this Article contained an error in Fig. 6b. In the top scattering process, while the positioning of both arrows was correct, the colours were switched: the first arrow was red and the second arrow was blue, rather than the correct order of blue then red.

Correspondingly, the caption to Fig. 6b incorrectly stated “The occurrence of scattering at the pyramid corners and/or at the merging points of two pyramidal domains, in the N region of the junction, makes quasiparticle trajectories emerging from a negative *p*_*y*_ lobe (blue arrow) on one side of the junction to couple with trajectories that probe the negative *p*_*y*_ lobe (red arrow) on the other side of the junction” instead of the correct “The occurrence of scattering at the pyramid corners and/or at the merging points of two pyramidal domains, in the N region of the junction, makes quasiparticle trajectories emerging from a negative *p*_*y*_ lobe (blue arrow) on one side of the junction to couple with trajectories that probe the positive *p*_*y*_ lobe (red arrow) on the other side of the junction.”

Also, for clarity, labels “π” and “0” have been added to the top and bottom scattering path, respectively.

This has now been corrected in both the PDF and HTML versions of the Article.

