# Supplementary material for: Induced unconventional superconductivity on the surface states of Bi2Te3 topological insulator
Source: Nat Commun. 2017 Dec 8;8:2019. doi: 10.1038/s41467-017-02069-z (PMC5722924; doi:10.1038/s41467-017-02069-z)
Supplement: Supplementary file 1 — Supplementary information [file 41467_2017_2069_MOESM1_ESM.pdf]

Supplementary Note 1. EFFECTIVE AREA OF A PLANAR JOSEPHSON JUNCTION

The effective area A_{eff} of a planar junction in the thin film limit $t < 2\lambda$, with t the film thickness and λ the London penetration depth, having a junction width, w, much smaller than the Pearl length, $\lambda_\perp = \lambda^2/t$, is well approximated by the following expression[1]

$$A_{\text{eff}} \approx wL + w^2/1.82 \,. \tag{1}$$

Here L is the distance between the electrodes (see Supplementary Figure 1). For junction widths larger than the Pearl length, Supplementary Equation (1) is not anymore valid and the effective area has to be computed numerically.

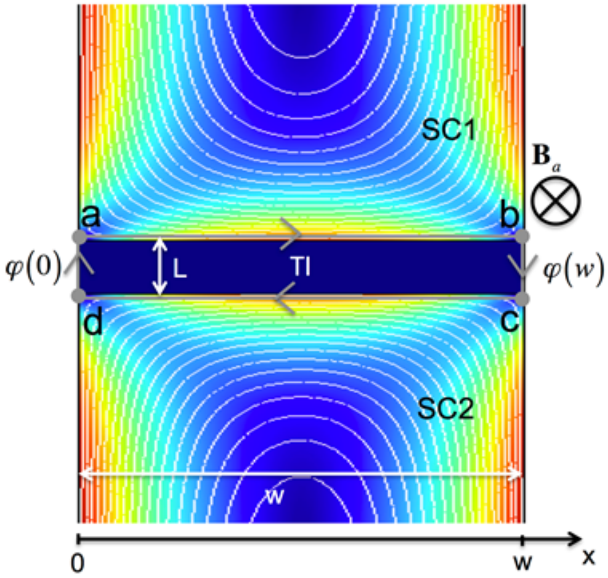

Supplementary Figure 1: **Supercurrent density distribution in a planar Josephson junction.** Sketch of two superconducting electrodes (SC1, SC2) contacted to a normal conducting channel (TI). The gray loop indicated the path integral used in Supplementary Equation (4). B_{a} is the externally applied magnetic field. The white lines indicate the current flow direction of the screening current and the color-coding in the electrodes reflects the amplitude of the screening current density (red: large, blue: small).

The effective area can be determined numerically as it follows.

First we calculate the Meissner screening currents in the superconducting electrodes by solving the Maxwell London equations on the junction geometry in the presence of an externally applied magnetic field:

$$\mu_0 \nabla \times (\lambda^2 \cdot \mathbf{j}) + \mathbf{B} = 0 \,; \qquad \nabla \times \mathbf{B} = \mu_0 \mathbf{J} \,. \tag{2}$$

Here μ_0 is the vacuum permeability, $\mathbf{j}$ is the supercurrent density in the electrodes, and $\mathbf{B}$ is the magnetic induction containing the externally applied field, B_a, and the field generated by the supercurrents.

Next we need to calculate the difference between the gauge invariant phase differences across the junction at the left edge $\phi(0)$ and right edge $\phi(w)$ of the junction (see Supplementary Figure 1). From the above calculated Meissner currents and resulting magnetic fields we can readily compute $\Delta\phi = \phi(w) - \phi(0)$ making use of the property that the superconducting phase is single valued in the electrodes:

$$\Delta\phi = \frac{2\pi}{\phi_0} \oint \mathbf{A} \cdot \partial \mathbf{l} + \mu_0 \lambda^2 \left[\int_a^b \mathbf{j} \cdot \partial \mathbf{l} + \int_c^d \mathbf{j} \cdot \partial \mathbf{l} \right] . \tag{3}$$

Here $\phi_0 = 2 \cdot 10^{-15}$ Vs is the superconducting flux quantum and $\mathbf{A}$ is the vector potential with $\mathbf{B} = \boldsymbol{\nabla} \times \mathbf{A}$. The closed path integral in the first term of the right hand side of the equation is taken along a loop enclosing the two edges of the junction at $x = 0$ and $x = w$. For the loop indicated in Supplementary Figure 1 the closed path integral corresponds to the total magnetic flux through the normal conductor in the junction. The line integrals in the second term of the right hand side of the Supplementary Equation (3) are taken only within the superconducting electrodes, i.e. from point a to b in the upper electrode, SC1, and from point c to d in the lower electrode, SC2 (see Supplementary Figure 1).

The effective area of the junction is finally obtained using the following standard expression:

$$A_\mathrm{eff} = \frac{\phi_0}{2\pi} \frac{\phi(w) - \phi(0)}{B_\mathrm{a}} . \tag{4}$$

In the above equation we assume a 2π periodic current phase relation along the junction.

For typical values of the London penetration depth ($\lambda \approx 100$ nm) for our 90 nm thick Al films[2], we obtain effective areas in the range $0.68 - 0.70\ \mu\mathrm{m}^2$, which is very close to the experimental value of $0.75\,\mu\mathrm{m}^2$. This strongly supports that a 2π periodic current phase relation mainly dominates the magnetic pattern of the critical current in our junctions.

Supplementary Note 2. EFFECTS OF THERMAL CYCLING AND OCCURRENCE OF $0 - \pi$ TRANSITIONS IN THE MAGNETIC PATTERN

We have measured a total of 14 devices, including Josephson junctions and dc SQUIDs (see Table 1 in the main text), all fabricated with flakes from the same Bi_2Te_3 film. 12 devices have been measured in a second cool down; 5 of them showed unconventional magnetic patterns, with a minimum at zero field.

Supplementary Figure 2 shows another example of a high field inverted pattern for a dc SQUID (SF8). In this case there are two different modulations of the critical current: one is determined by the SQUID loop and the other is determined by the magnetic response of the single junctions forming the SQUID, which gives a dip at zero magnetic field in the convolution of the maxima of the SQUID modulation (Supplementary Figure 2A). Supplementary Figure 2B shows the magnetic pattern of the SQUID at low fields.

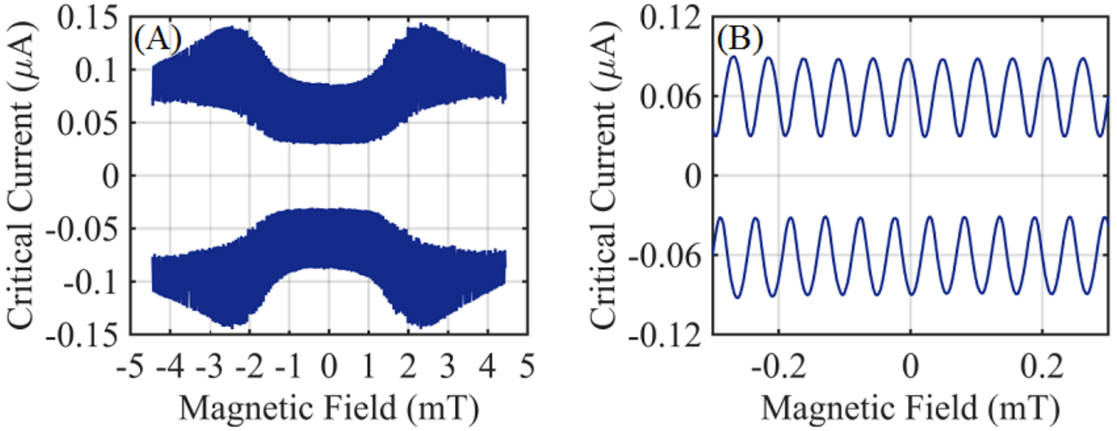

Supplementary Figure 2: **Critical current dependence as a function of the externally applied magnetic field of the SQUID FS8 at $T = 20$ mK.** Panel (**A**) clearly shows the feature of the magnetic response of the single junctions forming the SQUID, while the modulation due to the SQUID loop cannot be resolved on this scale (see text for details). The SQUID modulation becomes visible at low external magnetic fields (panel (**B**)).

Supplementary Note 3. MICROSCOPIC ORIGIN OF THE THERMAL CYCLING EFFECT

The thermal cycling effect has its origin in the role played by strain to tune the topological phase in TIs. In our devices, the complex interplay between the thermal properties of the substrate and the flake, the specifics of the interface between the Pt sticking layer and the Al, and the nanoscale dimensions of the devices have a fundamental role in creating plastic deformation at the TI nanogap separating the superconducting electrodes. The irreversible consequences of a compressive strain, induced by thermal cycling, manifest as peculiar buckling waves at the nanogap and a dramatic reduction of the Josephson current.

First principle calculations[3,4] have demonstrated that an in-plane tensile strain (pulling) leads to a out of plane compression where the quintuple layers are getting closer. An in plane compressive

strain (pushing) causes instead an out of plane expansion where the distance between quintuple layers is enhanced. The interquintuple interaction plays a dominant role in determining the topological phase: the consequence of an out of plane compression is a shift of the Dirac point closer to the valence band while an out of plane expansion leads to a gap opening at the Dirac point[3]. This argument is also used to explain why for example Sb_2Se_3 (with a larger c/a ratio compared to Bi_2Se_3) that is expected to be a TI in many ways, is instead a trivial insulator. It also tells that, in principle, strain can provide a tuning of the surface states of a topological insulator.

Strain is usually generated during the epitaxial growth of the material on the substrate, with lattice parameters different from those of the topological insulator. Recent reports have shown the tunability of the Dirac point with strain in thin topological crystalline insulator SnTe[5] and at grain boundaries in Sb_2Se_3 thin films[6].

Our experiment is quite different since our devices employ Bi_2Te_3 flakes exfoliated and transferred to a SiO_2/Si substrate, so a possible strain-related phenomenology cannot be attributed to the growth process.

The physics behind the peculiar transport properties of our devices is instead related to the enormous difference in the thermal expansion coefficient α of Bi_2Te_3 ($\approx 13.4 \times 10^{-6}$ °C^{-1}) and that of the SiO_2/Si substrate (0.5×10^{-6} °C^{-1}/2.4×10^{-6} °C^{-1}) where the flake is transferred. In our device configuration the Bi_2Te_3 is not grown on a SiO_2/Si substrate: the flake therefore will experience the huge difference in the thermal expansion coefficient only by the clamping to the substrate, which occurs through the patterning of the Al electrodes forming a nanometer sized gap junction.

At the first cool down, from room temperature to mK, the flake is prone to contract. However the Al electrodes will anchor the flake to the substrate making it experiencing the much smaller thermal coefficient of the SiO_2/Si. This leads to a tensile strain (pulling of the flake) which strongly concentrated in the part of the flake located at the nanogap. This important fact is related to the thermal expansion coefficient of Al ($\approx 23 \times 10^{-6}$ °C^{-1}) not much different from that of the Bi_2Te_3. For a good bonding between the Al and the flake, as the one provided by the Pt sticking layer, one can consider the Al electrodes and the flake under them as forming a quite homogenous material with a thickness more than twice that of the flake (the thickness of Al is roughly twice that of the flake). In this way most of the strain, provided by the Al electrodes and locking the flake to the substrate, is located at the thin Bi_2Te_3 nanogap, with a smaller thickness compared to that of the composite material Al/flake.

Since the tensile strain concentrates in the nanogap region, in this part of the flake the Dirac

node is shifted towards the valence band which makes the Bi_2Te_3 channel effectively more doped.

During the warming up of the sample the Bi_2Te_3 at the nanogap undergoes instead a compressive strain which opens a gap at the Dirac point. This compressive strain induces plastic deformation (the sample properties are completely changed after thermal cycling) and, if it overcomes a critical strain, can lead to a buckling of the Bi_2Te_3 channel forming the nanogap.

Supplementary Figure 3a shows a SEM top picture of one of the sample discussed in our paper. This specific picture does not show, very clearly, possible anomalous features of the flake inside

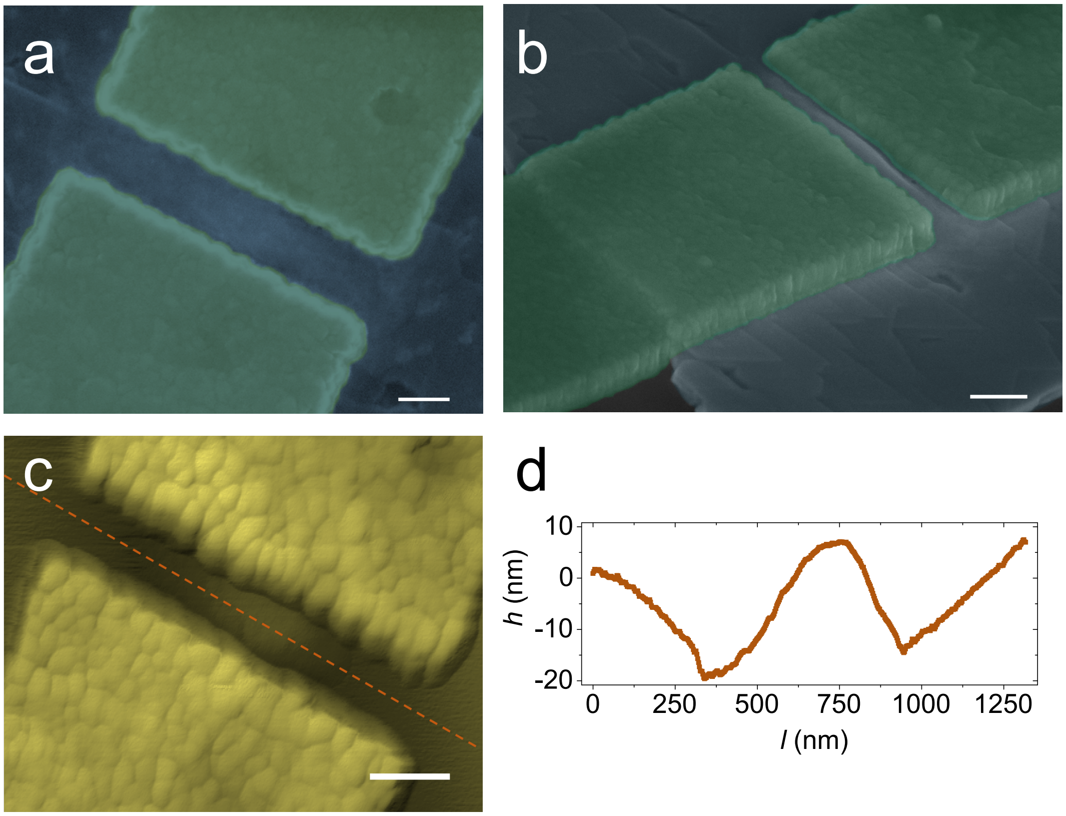

Supplementary Figure 3: **Colored SEM and AFM images for a junction showing a 0-π magnetic pattern.** This device is one of those discussed in our main manuscript (Pt interlayer). (**a**) SEM top view (scale bar: 200 nm); (**b**) tilted view (scale bar: 200 nm), green color represents the electrodes; (**c**) AFM height image (scale bar: 150 nm); (**d**) height profile along the junction.

the nanogap, except a region with a slightly brighter color. However by taking a SEM picture at an angle, shown in Supplementary Figure 3b, the brightness at the nanogap clearly manifests as a buckling feature[7]. To further evaluate the characteristics of such a buckling, we have performed AFM analysis to resolve the shape of the deformation inside the nanogap. Supplementary Figure 3c shows an AFM picture of the same junction, while Supplementary Figure 3d a line scan (orange

dashed line) taken inside the nanogap. The line scan has a very clear sinusoidal-like shape, a distinctive feature of a bucking phenomenon induced by compressive strain.

A. Role of the interlayer material: comparison between transport properties of Al/Pt/Bi_2Te_3/Pt/Al and Al/Ti/Bi_2Te_3/Ti/Al

In our previous works[8,9], we have studied the transport properties of Al/Pt/Bi_2Se_3/Pt/Al and Al/Ti/Bi_2Se_3/Ti/Al where the flakes were exfoliated from single crystals. The comparison showed much larger critical current densities and $I_C R_N$ product for devices obtained with a Pt interlayer, both facts pointing towards a higher interface transparency barrier (I) (see Supplementary Figure 4), instrumental to observe Majorana bound state physics.

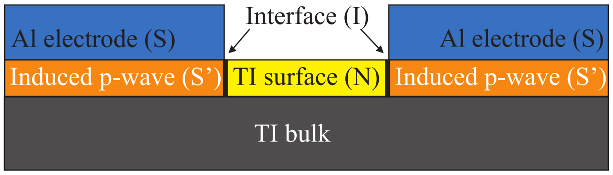

Supplementary Figure 4: **Cross section schematics of the effective device under consideration.** The transport properties can be assimilated to those of a S'INIS' Josephson junction where S' represent the induced chiral p-wave superconductor in the TI.

We have repeated this experiment on the Bi_2Te_3 flakes of this work, with the peculiar triangular morphology, by using a Ti interlayer.

Supplementary Table 1 summarizes the main transport properties of the junctions fabricated using both Pt and Ti. We confirm the general trend: the J_C and $I_C R_N$ values for the Pt interlayers are on average more than 5 times higher than those obtained with Ti. The same trend, higher values for Pt junctions, is observed for the average transparencies of the barrier I. This comparison is made at the first cool down.

	J_C (μA/μm)	$I_C R_N$ (μeV)	τ (transparency)
Rough flakes, **Pt** interlayer	2.7 ± 0.8	79 ± 19	0.67 ± 0.05
Rough flakes, **Ti** interlayer	0.41 ± 0.14	13 ± 3	0.49 ± 0.01

Supplementary Table 1: **Main parameters extracted from the IVCs of the Bi_2Te_3 junctions with different interlayer metals**.

Supplementary Figure 5 compares the conductance spectra, obtained by differentiating the current voltage characteristics (IVCs), of various junctions fabricated with Ti interlayer, with those obtained with a Pt interlayer. The curves of Supplementary Figure 5b (Ti interlayer) clearly show a dip at $V \approx 230\,\mu$eV that we have identified as $2\Delta_{S'}$, where $\Delta_{S'}$ is the induced gap into the TI[10,11]. In the spectra of Supplementary Figure 5b no other dips can be clearly identified. Supplementary Figure 5d shows instead various spectra relative to the IVCs of junctions fabricated with Pt interlayer. Also in this case we can identify a well developed dip at $V \approx 230\,\mu$eV (much more pronounced than the Ti case due to the higher transparency τ of the barrier I) that we correlate with $2\Delta_{S'}$, where again $\Delta_{S'}$ is the induced gap (this correlation is unambiguous because of the peculiar temperature dependence of $2\Delta_{S'}$[10,11]).

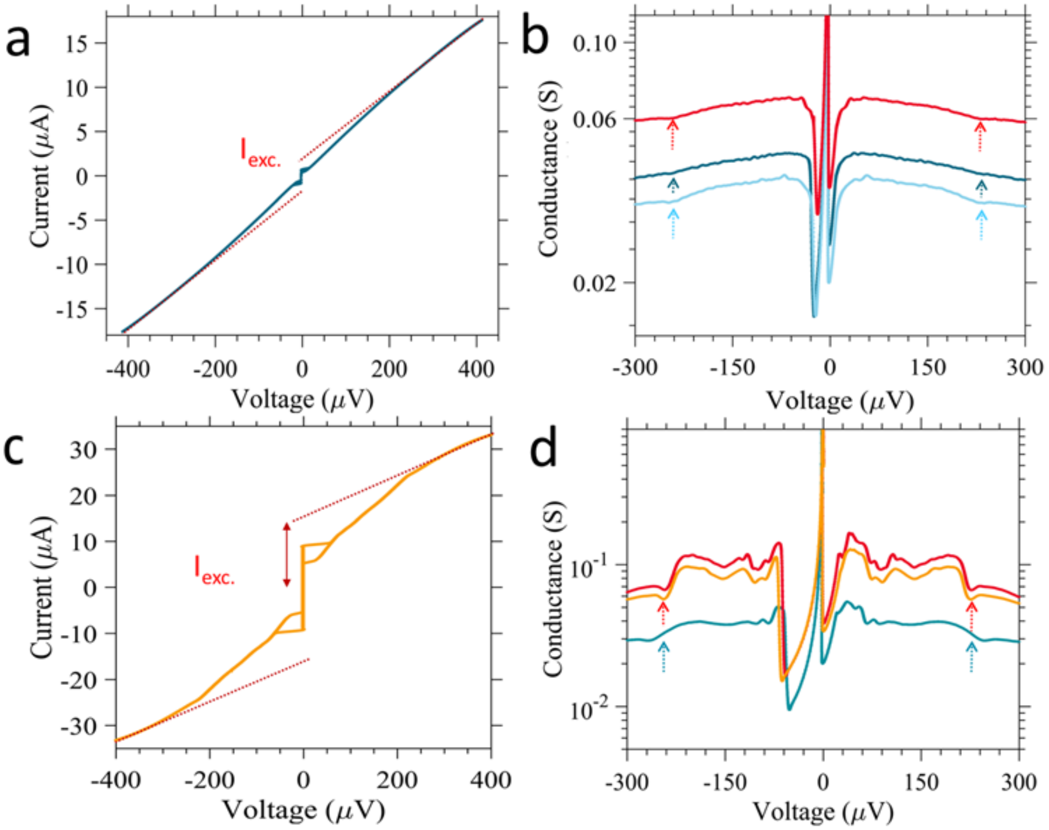

Supplementary Figure 5: **IVCs and conductance spectra for different interlayer metals.** IVCs of the Bi_2Te_3 junctions with (**a**) - Ti and (**c**) - Pt interlayer metal; the dashed line in both graphs represents linear fit to extract the excess current. Conductance spectra for three junctions with (**b**) - Ti and (**d**) - Pt interlayer metals; the arrows indicate the deeps corresponding to $2\Delta_{S'}$.

However the conductance curves of the junctions with Pt have many more dips at different voltages, associated to multiple Andreev reflections and that are made visible because of the higher

transparencies interfaces I, as we find in our experiment[11]. To summarize these measurements we observe 1) the interface Bi_2Te_3/Pt/Al and Bi_2Te_3/Ti/Al have the same transparency since the induced gap $\Delta_{S'} \approx 115\,\mu$eV is *exactly the same for both kind of junctions* and 2) the difference in the J_C values, $I_C R_N$ and transparency τ has to be related to the higher transparent interfaces I, between the part of the flake in the nano-channel and that under the electrodes, for the Pt junctions. The magnetic pattern of the Josephson current for both Pt and Ti junctions are Fraunhofer-like at the first cool down. However at the second cool down the junctions with Ti interlayer are not affected by the thermal cycle. The IVCs and the magnetic Fraunhofer pattern do not have any variation. We have cooled down the junctions from room to 20 mK temperature 3 times and the transport properties are completely preserved. This fact is in line with the results obtained by other groups working with 3DTI and using Ti as sticking layer.

What makes the junctions with Pt interlayers so different from the Ti?

We tend to exclude an origin related to a different chemistry between Pt and Ti while bonding to the Bi_2Te_3. This is because the values of the induces gap $\Delta_{S'}$ are identical, which is a consequence of very similar interface transparency.

Instead we will show that the completely different behavior between Ti and Pt junctions is related to different growth habits of the two metals on Bi_2Te_3.

Supplementary Figure 6a shows an AFM picture of a 3 nm Pt grown on a Bi_2Te_3 flake. The image shows a uniform layer that nicely wets the Bi_2Te_3 flake smoothening edges and corners. The growth habits of a 3 nm Ti film are instead quite different (see Supplementary Figure 6b). The Ti does not wet forming tiny grains (on average 20 nm in diameter) on the surface of Bi_2Te_3. As a result the morphology of the Al film, which grows on top of the Ti is much more granular (data not shown), compared to the growth on Pt.

The Al electrodes have the role of clamping the flake to the substrate so as to experience a tensile and compressive strain. For this to happen the Al and the flake underneath need to behave like almost the same material. The wetting properties of the Pt on the Bi_2Te_3 appear to promote an excellent Al adhesion and therefore an effective clamping of the flake to the substrate. The granular growth of the Ti interlayer on Bi_2Te_3 instead does not seem to be as effective in this respect. While the interface properties of the Al and Bi_2Te_3 through the Ti are very similar to the Pt junctions, the granularity of the Ti sticking layer makes the Al and the Bi_2Te_3 flake not as strongly connected to each other to behave as a homogenous materials. In this way no plastic

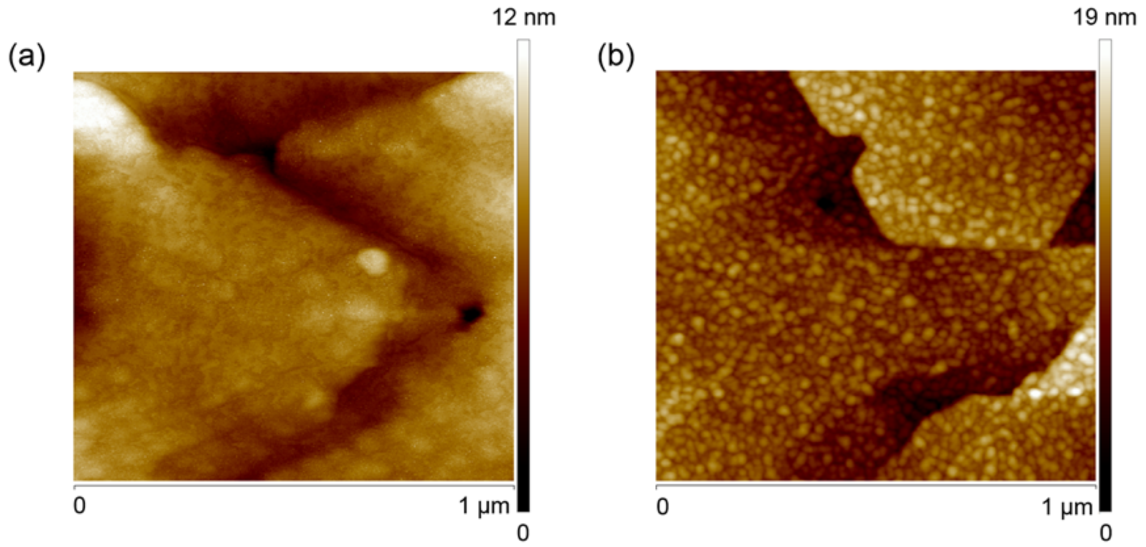

Supplementary Figure 6: **Growth habits of Pt and Ti on a Bi_2Te_3 flake.** AFM images of a Bi_2Te_3 flake covered (**a**) with 3 nm Pt and (**b**) with 3 nm Ti.

deformations are induced. Supplementary Figure 7b shows a typical top view SEM picture for one of the measured junction after several cool down; Supplementary Figure 7a is a SEM image of the same device taken with a tilted angle. None of the images show signature of buckling wave that instead we have detected in junctions with Pt interlayer.

It is worth mentioning what could be the origin of the much higher critical current densities we observe in junctions with Pt interlayer compared with those with Ti at the first cool down.

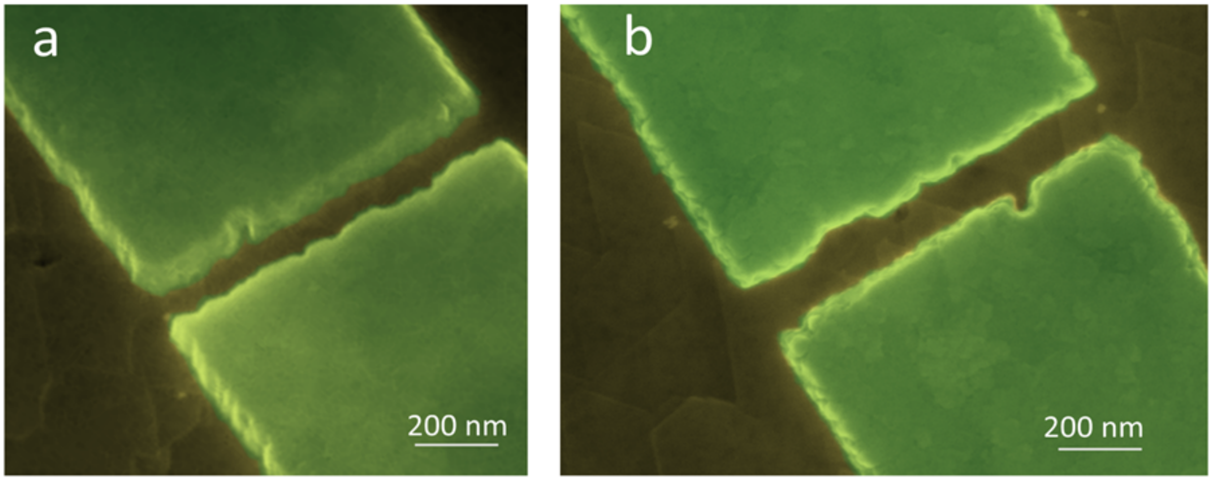

Supplementary Figure 7: **Colored SEM images for a junction with Al electrodes and Ti interlayer.** (**a**) Tilted view; (**b**) side view. The electrodes are represented in light green color.

We believe it is a consequence of the occurrence of a tensile strain experienced by the flake in the nanogap.

The origin of the barrier I between the flake under the Al/Pt(Ti) electrodes and the one in the nanogap is not clearly established. One can possibly expect some doping from the metal, which shifts the Fermi energy of the flake under the electrode, compared to the one in the nanogap. The occurrence of a tensile strain at the nanogap while cooling the nanodevice, shift the Dirac point toward the valence bands making it more doped. Since the effect of strain is much more dominant in junctions with Pt, one can expect a better Fermi level matching between the two parts of the flake, under the electrodes and at the nanogap, resulting in a higher transparency barrier and higher J_C values.

Supplementary Note 4. TWO DIMENSIONAL MAGNETO-FINGERPRINTS IN Bi_2Te_3 CHANNELS

In the paper by Kandala et al.[12], the authors study the magneto transport of Bi_2Se_3 channels, where the films have a typical growth with pyramidal domains. The authors reveal signatures of Aharonov-Bohm (AB) orbits, manifesting as periodic magneto conductance fluctuations. The length scale of the orbits corresponds to the typical perimeter of triangular terraces found on the surface of these thin film devices. They conclude that the periodic magneto-fingerprint arises from coherent scattering of electron waves from the step-edges. To demonstrate that this scenario is also valid for the Bi_2Te_3 flakes, used in our work, we have preformed magneto transport measurements down to 20 mK temperature on devices with a Hall configuration.

Supplementary Figure 8a shows the longitudinal resistance between the two voltage probes with a distance of 0.5 μm (the classical quadratic magneto-resistance background has been subtracted for clarity) in the range of magnetic field 1-3 T for various temperatures. From the Fourier transform of the curves, Supplementary Figure 8b, we clearly observe a peak around 2.9 T^{-1} which corresponds to an area of 0.012 μm^2 in line with the morphology of our flakes and in complete agreement with the values reported in Ref. 12 for a device of similar dimensions as ours. Moreover we clearly see that the amplitude of the magnetoresistance oscillations decreases with temperature (inset of Supplementary Figure 8b) in a similar fashion as in Ref. 12. This strongly suggests that also in our case the periodic magneto-resistance oscillations arise from coherent scattering of electron wave from the corners and/or step-edges where two pyramidal domains merge.

It is also worth discussing if one can find any apparent correlation, between the orientation of the

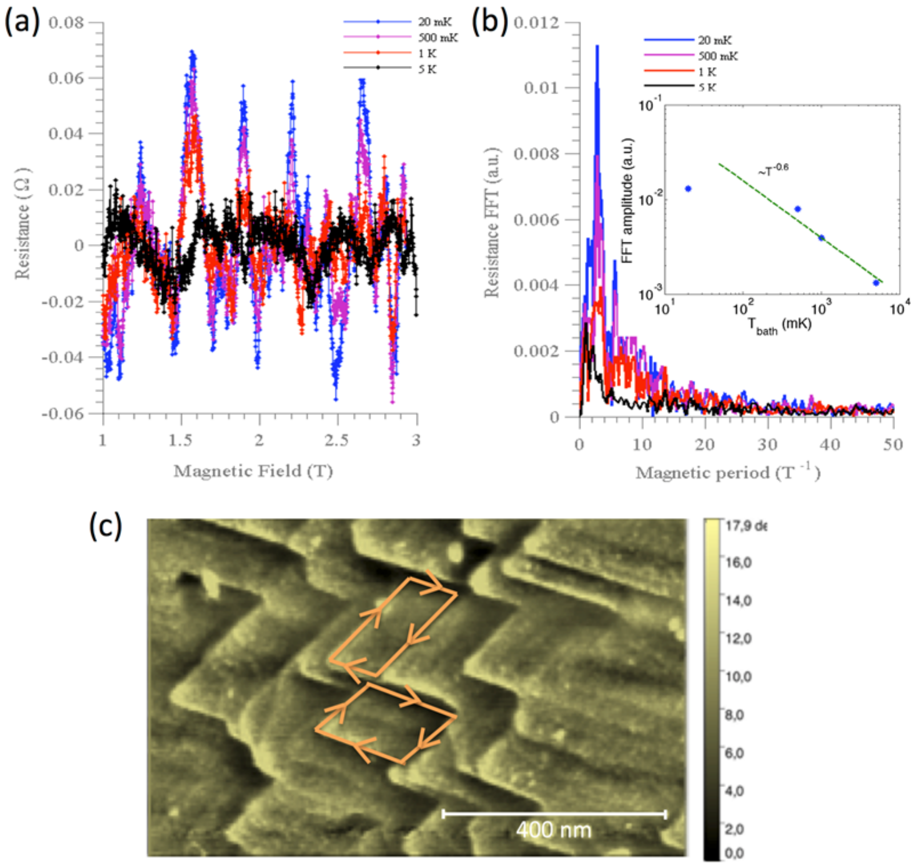

Supplementary Figure 8: **Two dimensional magneto-fingerprints in Bi_2Se_3 channels.** (**a**) Longitudinal resistance as a function of magnetic field for a channel length of 0.5 μm at different temperatures. (**b**) Fourier transform of the curves of panel (a). The inset shows the Fourier transform amplitude at 2.9 T^{-1} as a function of temperature. The data have a typical power law dependence (with exponent -0.6). (**c**) Typical surface morphology of the flakes used in the experiment. The orange lines delimitate two possible Arhonov-Bhom orbits with approximately the same area.

nano-pyramidal domains and that of the electrodes, with the occurrence of unconventional magnetic field pattern related to 0 and π trajectories inside the Bi_2Te_3 nanochannel. We have investigated by SEM all the junctions having a 0-π transition and we do not find any clear correlation between the orientation of the nano-pyramidal domains and that of the electrodes. However this is not surprising if one considers that the basic mechanism to get an inverted magnetic field pattern, with a minimum of the critical current at zero external field, is the occurrence of 0 and π facets within the same nanogap. In our flakes the occurrence of AB oscillations supports preferential trajectories of electrons and holes along the edges of the triangles. Supplementary Figure 9 shows

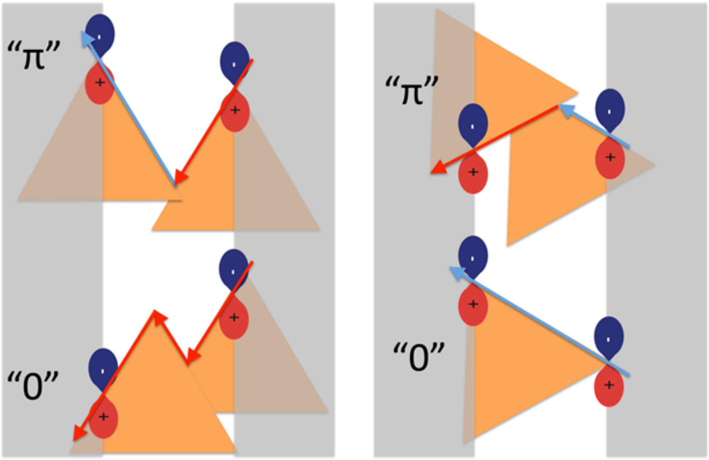

Supplementary Figure 9: **Schematic pictures of a Josephson junction with different orientation of the pyramidal domains in the nanogap with respect to the Al electrodes.** In the left panel, the main direction of the pyramidal domains is aligned parallel to the Al electrodes, while it is perpendicular to them in the right panel. In the figure, 0 and π trajectories can be found in both cases.

a sketch corresponding to two extremes cases where the main alignment direction of the pyramidal domains is parallel (Supplementary Figure 9a) or perpendicular (Supplementary Figure 9b) to the electrodes. We see that in both cases one can find 0 and π trajectories, which would lead to an inverted Fraunhofer pattern.

Supplementary Note 5. PERIODICITY OF A MAGNETIC PATTERN WITH REGULAR AND RANDOM DISTRIBUTED 0-π FACETS.

The magnetic patter of a regular 0-π faceted Josephson junction is strongly modified compared to a Fraunhofer-like. Supplementary Figure 10 shows the $I_C(B)$ for a junction formed by a total of 10 equal in size 0 and π facets carrying the same current[17]. The absolute maximum of the pattern is found at $5\phi_0$. This is quite easy to understand since one requires a ϕ_0 to reverse the phase sign of every π facet. However one can still find the periodicity connected to the total junction's width in the position of the minima which happen at multiple of ϕ_0 (see Supplementary Figure 10). For a random distribution of facets the absolute maximum will always occur at finite field (which will depend on the microscopic distribution of 0-π facets), while at zero external field one can find a local maximum (not the absolute maximum) or a local minimum again dependent on the details of the facets microscopic distribution.

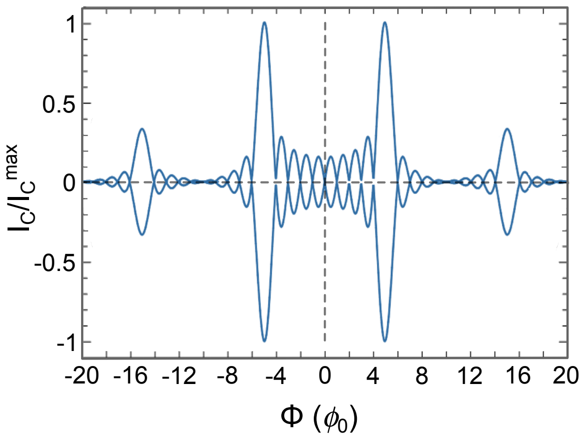

Supplementary Figure 10: **Simulated magnetic flux dependence of the Josephson current for an array of 10 alternating 0 and π facets.**

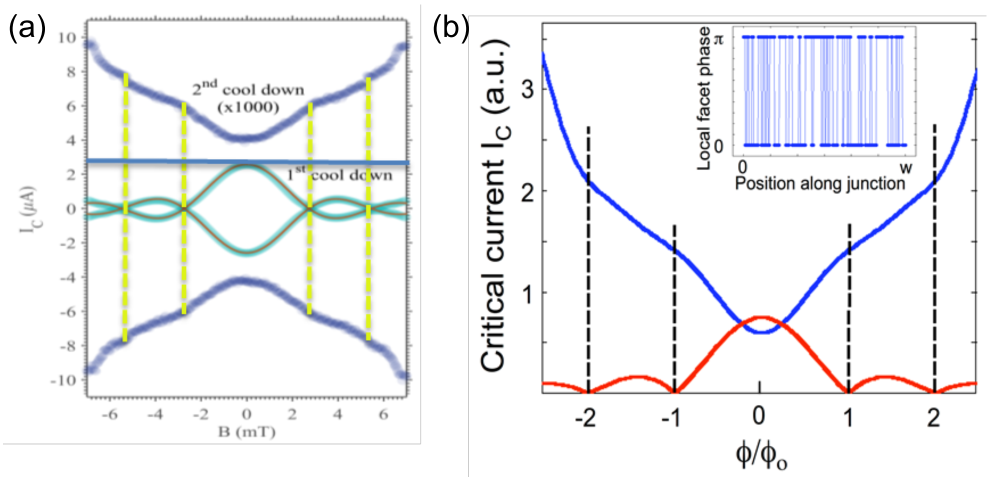

Supplementary Figure 11: **Magnetic field dependence of a typical Bi_2Te_3 junction compared with that of a simulated array of random distributed 0 and π facets.** (**a**) Critical current I_C dependence on the externally applied magnetic field B for one of our junctions at 20 mK, before (cyan) and after (blue) the thermal cycle. At the first cool down the $I_C(B)$ dependence shows a conventional Fraunhofer dependence. After the thermal cycle (blue points), the critical current is dramatically reduced (the data points are multiplied by a factor 1000 for clarity), and a dip at B=0 appears. (**b**) Numerically calculated $I_C(B)$ (blue curve) for a random distribution of 0 and π facets (see inset) compared with a conventional Fraunhofer type dependence calculated considering a uniform current distribution.

The periodicity connected to the size of the junction can be still identified as local minima, local maxima or change in slope in the $I_C(B)$. As an example in Supplementary Figure 11(b) we show the computed magnetic field dependence $I_C(B)$ for a uniform current distribution (red curve) compared with a random distribution of 0 and π facets (blue curve).

For the random case we can clearly identify change of slopes at ϕ_0 and $2\phi_0$ (indicated by the dashed lines) which corresponds to the periodicity determined by the width of the junction. Indeed such a scenario qualitatively reproduces what we observe in our experiment (see Supplementary Figure 11(a)) and that is discussed in the manuscript.

Supplementary Note 6. FIT OF THE $I_C(T)$ DEPENDENCE WITH A SINIS MODEL

The full theory of the Josephson effect in SINIS structures with chiral p-wave (p_x + ip_y) superconducting leads is still lacking. In Ref. 13, Sawa et al have considered a SINIS system with p_x + ip_y superconducting leads and diffusive normal part, numerically solved Usadel equations and determined the $I_C(T)$ dependence. This dependence turns out to be qualitatively very similar to that of an usual SINIS junction with s-wave leads. Moreover, in the limit of highly transparent insulating barriers $I_C(T)$ dependence for s-wave and p-wave leads are found to be essentially identical. In Ref. 14 the authors have considered a SINIS structure with chiral p-wave leads and short ballistic normal metal. These authors have also found that for perfectly transmitting barriers both p_x + ip_y and s-wave pairing symmetries lead to the same $I_C(T)$ dependence. In view of these previous works, we have modeled our highly transparent junctions measured during the first cool down as S'INIS' structures (where S' is the proximity induced superconductor) with conventional s-wave superconducting leads, for which a simple analytical formula for the Josephson current exists in the clean ballistic limit[15]. The same approach has been adopted in an earlier work[16].

For our S'INIS' junctions, we consider the limit of wide junction, $k_F w \gg 1$, and use the following expression for the critical current

$$I_J(\phi) = \frac{4ek_BT}{\hbar}\frac{k_F w}{\pi}\sin\phi \sum_{\omega_n>0}\int_0^1 d\mu \frac{t_1 t_2}{\sqrt{Q\left(\phi, \sqrt{1-\mu^2}\right)}} . \tag{5}$$

Here $\mu = \sin\theta$, where θ is the angle between the velocity of an electron flying out of a lead and the shortest line connecting the two leads,

$$t_1 = \frac{D_1}{2-D_1}\ , \qquad t_2 = \frac{D_2}{2-D_2} \tag{6}$$

are the effective Andreev transparencies of the barriers[16], D_1 and D_2 are the usual barrier transparencies in the normal state. In Eq. (5), the function Q is defined as

$$Q\left(\phi,\sqrt{1-\mu^2}\right)=\left[t_1t_2\cos\phi+\left(1+(1+t_1t_2)\frac{\hbar^2\omega_n^2}{\Delta^2}\right)\cosh\left[2\omega_n t_0(\mu)\right]+\right.$$
$$\left.+(t_1+t_2)\frac{\hbar^2\Omega_n\omega_n}{\Delta^2}\sinh\left[2\omega_n t_0(\mu)\right]\right]^2-(1-t_1^2)(1-t_2^2)\frac{\hbar^4\Omega_n^4}{\Delta^4}\;, \tag{7}$$

$$\omega_n=\frac{\pi k_{\mathrm{B}}T(2n+1)}{\hbar}\;, \tag{8}$$

$$\hbar\Omega_n=\sqrt{\hbar^2\omega_n^2+\Delta^2}\;, \tag{9}$$

ω_n are the Matsubara frequencies, and $t_0(\mu)$ is the angular dependent average flight time of an electron between the leads for a given Fermi speed v_{F}, mean free path l_{e}, the separation between the leads L, and

$$t_0(\mu)=\frac{l_e}{(1-\mu^2)v_{\mathrm{F}}\left(\sqrt{1+\frac{l_{\mathrm{e}}^2}{L^2(1-\mu^2)}}-1\right)}\;. \tag{10}$$

Supplementary Equation (10) may be viewed as an interpolation between the clean limit ($l_{\mathrm{e}}\gg L$), in which case

$$t_0(\mu)=\frac{L}{v_{\mathrm{F}}\sqrt{1-\mu^2}}\;, \tag{11}$$

and the diffusive limit ($l_{\mathrm{e}}\ll L$) , where t_0 does not any more depend on the angle and reads

$$t_0=\frac{2L^2}{v_{\mathrm{F}}l_{\mathrm{e}}}\;. \tag{12}$$

For the normal state resistance in this model we find

$$\frac{1}{R}=\frac{e^2}{\pi\hbar}\frac{k_{\mathrm{F}}w}{\pi}\frac{1}{\frac{1}{D_1}+\frac{1}{D_2}-1+\frac{2L}{\pi l_{\mathrm{e}}}}\;. \tag{13}$$

The model outlined above differs from the original one[15] in two ways. Since we are dealing with a two dimensional normal layer (while in Ref. 15 a three dimensional normal metal was considered), we have replaced the integral over the two transverse components of the wave vector, k_y and k_z, by the corresponding one-dimensional integral over k_y, i.e. we have replaced $dk_ydk_z/(2\pi)^2$ by $dk_y/2\pi h$, where h is the flake thickness. Second, we have introduced finite mean free path into the expression for the Josephson current in a phenomenological way.

Namely, we have replaced the ballistic expression for the flight time, Supplementary Equation (11), by a more complicated one containing the mean free path, Supplementary Equation (10). We

have verified that this replacement correctly reproduces the known expression for the Josephson current of a short diffusive SINIS junction.

We have performed a fit on a selection of the junction listed in Table 1 (see main text), which covered a wide range in the magnitude of the critical current values (see Supplementary Figure 12). We used a value for the S' superconducting gap of $\Delta_{S'} \approx 125\,\mu$eV, which is close to the value of the Al superconducting gap. The Fermi velocity of the electrons has be chosen to be $v_F = 3.5 \cdot 10^5$

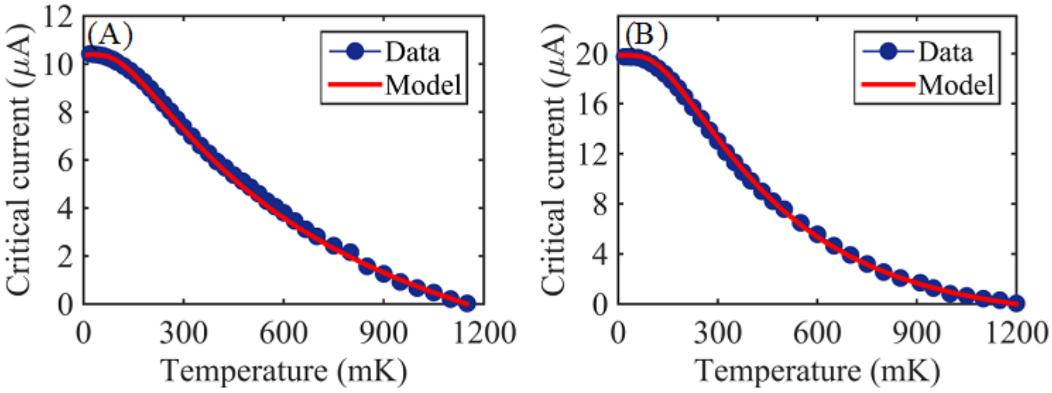

Supplementary Figure 12: **Critical current as a function of the temperature at the first cool down.** The measured $I_C(T)$ dependencies are for samples MS1 (panel **A**) and FS4 (panel **B**). The red line is the best fit, considering a quasi-ballistic model (Supplementary Equations (5) and (7)). The fit parameters are listed in Supplementary Table 2.

m s^{-1}, in agreement with literature, and the fitting parameters are: the Fermi energy, E_F, the mean free path, l_e, and the transparencies of the barriers, which we assumed to be identical, $D_1 = D_2 = D$. The theoretical value of the normal state resistance, R_{th}, has been determined from Supplementary Equation (13) and the Thouless energy was estimated as $E_{Th} = h v_F l_e / L^2$.

The extracted values of the mean free path (l_e) are typically comparable to the junction length, thus confirming a picture of an intermediate, between ballistic and diffusive, transport regime. The scattering centers are probably the edges of the pyramidal domains of the film, as we have discussed in the main text.

It is known[16] that the bulk of the flake should behave as a resistive shunt. From the expression

$$\frac{1}{R_{sh}} = \frac{1}{R_{exp}} - \frac{1}{R_{th}} \tag{14}$$

we have extracted the effective shunt resistances of the devices. In Supplementary Equation (14) R_{th} is the prediction of Supplementary Equation (13) and R_{exp} is the resistance measured in the experiment. The sheet resistances of the films associated with the bulk transport, $R_\square = R_{sh} w/L$,

vary in the range between 150 and 550 $\Omega/\Box$. The corresponding resistivities, defined as $\rho = R_\Box h$, are shown in Supplementary Table 2 and change between 1000 and 5000 $\mu\Omega$cm, which is about one order of magnitude larger than the bulk resistivity reported in Ref. 16, which probably indicates a lower level of doping in our material. We believe that the scattering of the extracted values of the bulk resistivity between different devices is mostly caused by the uncertainty in the geometry of the samples, but it may also be explained by slightly different levels of doping.

**Device**	l_e (nm)	D	E_F (meV)	E_{Th} (μeV)	R_N^{th} (Ω)	R_{sh} (Ω)	$R_\Box$ (Ω)	ρ ($\mu\Omega$cm)
**JM1**	140	.99	106	363	566	101	152	1200
**JM10**	130	.98	140	341	134	47	236	1900
**SM4**	100	.98	172	954	45	27	543	4300
**SM1**	95	.98	213	920	37	25	502	4000
**SF4**	35	.94	210	392	22	3.5	209	1670

Supplementary Table 2: **Parameters extracted from the fit of the $I_C(T)$.** The mean free path l_e, the transparency of the barriers D (assuming the two barriers are identical) and the Fermi energy E_F are the free parameters of the fit. From these parameters we derived the Thouless energy E_{Th}, the expected resistance above the gap R_N^{th}, the calculated shunt resistance of the flake R_{sh}, and the corresponding sheet resistance $R_\Box$ and resistivity ρ. The induced gap at zero temperature was taken to be $\Delta(0) = 125\,\mu$eV and at higher temperatures the standard BCS gap temperature dependence has been used.

SUPPLEMENTARY REFERENCES

[1] Rosenthal, P. A., Beasley, M. R., Char, K., Colclough, M. S. & Zaharchuk, G. Flux focusing effects in planar thin-film grain-boundary Josephson junctions. *Appl. Phys. Lett.* **59**, 3482 (1991).

[2] Romijn, J., Klapwijk, T. M., Renne, M. J. & Mooij, J. E. Critical pair-breaking current in superconducting aluminum strips far below T_c. *Phys. Rev. B* **26**, 3648-3655 (1982).

[3] Liu, W. et al. Anisotropic interactions and strain-induced topological phase transition in Sb_2Se_3 and Bi_2Se_3. *Phys. Rev. B* **84**, 245105 (2011).

[4] Young, S. M. et al. Theoretical investigation of the evolution of the topological phase of Bi_2Se_3 under mechanical strain. *Phys. Rev. B* **84**, 085106 (2011).

[5] Zeljkovic, I. et al. Strain engineering Dirac surface states in heteroepitaxial topological crystalline insulator thin films. *Nat. Nanotechnol.* **10**, 849-853 (2015).

[6] Liu, Y. et al. Tuning Dirac states by strain in the topological insulator Bi_2Se_3. *Nature Phys.* **10**, 294-299 (2014).

[7] Bowden, N., Brittain, S., Evans, A. G., Hutchinson, J. W., & Whitesides, G. M. Spontaneous formation of ordered structures in thin films of metals supported on an elastomeric polymer. *Nature* **393**, 146-149 (1998).

[8] Galletti, L. et al. Influence of topological edge states on the properties of Al/Bi_2Se_3/Al hybrid Josephson devices. *Phys. Rev. B* **89**, 134512 (2014).

[9] Galletti, L. et al. Josephson effect in Al/Bi_2Se_3/Al coplanar hybrid devices. *Physica C* **503**, 162 (2014).

[10] Kjaergaard, M. et al. Transparent Semiconductor-Superconductor Interface and Induced Gap in an Epitaxial Heterostructure Josephson Junction. *arXiv:1607.04164v2* (2016)

[11] Galletti, L. et al. High-Transparency Al/Bi_2Te_3 Double-Barrier Heterostructures. *IEEE Trans. Appl. Supercond.* **27**, 1-4 (2017).

[12] Kandala, A., Richardella, A., Zhang, D., Flanagan, T. C., & Samarth, N. Surface-sensitive two-dimensional magneto-fingerprint in mesoscopic Bi_2Se_3 channels. *Nano Lett.* **13**, 2471-2476 (2013).

[13] Sawa, Y., Yokoyama, T., Tanaka, Y. & Golubov, A. A. Quasiclassical Green's function theory of the Josephson effect in chiral p-wave superconductor/diffusive normal metal/chiral p-wave superconductor junctions. *Phys. Rev. B* **75**, 134508 (2007).

[14] Tkachov, G. & Hankiewicz, E. M. Helical Andreev bound states and superconducting Klein tunneling in topological insulator Josephson junctions. *Phys. Rev. B* **88**, 075401 (2013).

[15] Galaktionov, A. V. & Zaikin, A. D. Quantum interference and supercurrent in multiple-barrier proximity structures. *Phys. Rev. B* **65**, 184507 (2002).

[16] Veldhorst, M. et al. Josephson supercurrent through a topological insulator surface state. *Nat. Mater.* **11**, 417-421 (2012).

[17] Smilde, H. J. H. et al. d-Wave-Induced Josephson Current Counterflow in $YBa_2Cu_3O_7$/Nb Zigzag Junctions. *Phys. Rev. Lett.* **88**, 057004 (2002).
